# Intra-aortic balloon pump in patients undergoing VA-ECMO: an analysis of the Chinese Extracorporeal Life Support Registry

**DOI:** 10.1186/s13054-024-04878-3

**Published:** 2024-03-21

**Authors:** Liangshan Wang, Xing Hao, Chenglong Li, Haixiu Xie, Feng Yang, Hong Wang, Zhongtao Du, Xiaotong Hou

**Affiliations:** Center for Cardiac Intensive Care, Beijing Anzhen Hospital, Capital Medical University, Beijing, People’s Republic of China

Venoarterial extracorporeal membrane oxygenation (VA-ECMO) has been increasingly used to treat refractory cardiogenic shock (CS) or cardiac arrest (CA) over the past decades. Peripheral VA-ECMO increases left ventricular (LV) afterload, potentially impairing myocardial recovery and leading to poor outcomes. Intra-aortic balloon pump (IABP) has been suggested as an approach to unload LV in patients supported by VA-ECMO [1]. However, the effectiveness of IABP combined with VA-ECMO remains controversial [2–4]. Using the data from the Chinese Extracorporeal Life Support (CSECLS) registry, we aimed to evaluate in-hospital outcomes in CS patients who received VA-ECMO with or without IABP.

The CSECLS registry is a voluntary database that collects information on ECMO use, complications, and outcomes in adults and children from more than 112 member centers in China. Data were collected using a standardized electronic reporting sheet submitted on the organization's website. We included adults (≥ 18 years) who received femoro-femoral VA-ECMO with IABP (IABP group) or without IABP (non-IABP group) from January 1, 2017, through August 31, 2022. We excluded patients received central cannulation or other LV unloading strategies. The primary outcome was in-hospital mortality. Secondary outcomes included survival to ECMO weaning, continuous renal replacement therapy (CRRT), cannulation site bleeding, and limb ischemia. This study was approved by the Research Ethics Board of the Beijing Anzhen Hospital (2021020X).

A total of 4755 VA-ECMO patients were included into the analysis, of whom 1147(30.4%) were in the IABP group, and 3308 (69.6%) were in the non-IABP group. The characteristics of the patients are presented in Fig. 1A. Patients in the IABP group were older (58 years vs 55 years), were more often male (75.1% vs 69.3%), were heavier (69 kg vs 67 kg), and were more likely to have acute myocardial infarction (AMI) as the primary cause of CS (65.4% vs 33.5%) (*p* < 0.05 for all). Patients in the IABP group had slightly higher pH at cannulation (7.30 vs 7.25, *p* < 0.001), were less likely to have pre-ECMO arrest (34.7% vs 40.7%, *p* < 0.001), and were less likely to receive ECPR (13.3% vs 18.7%, *p* < 0.001). The time on VA-ECMO support was longer in the IABP group as compared to the non-IABP group (4.4 days vs 3.0 days, *p* < 0.001).

**Fig. 1:**
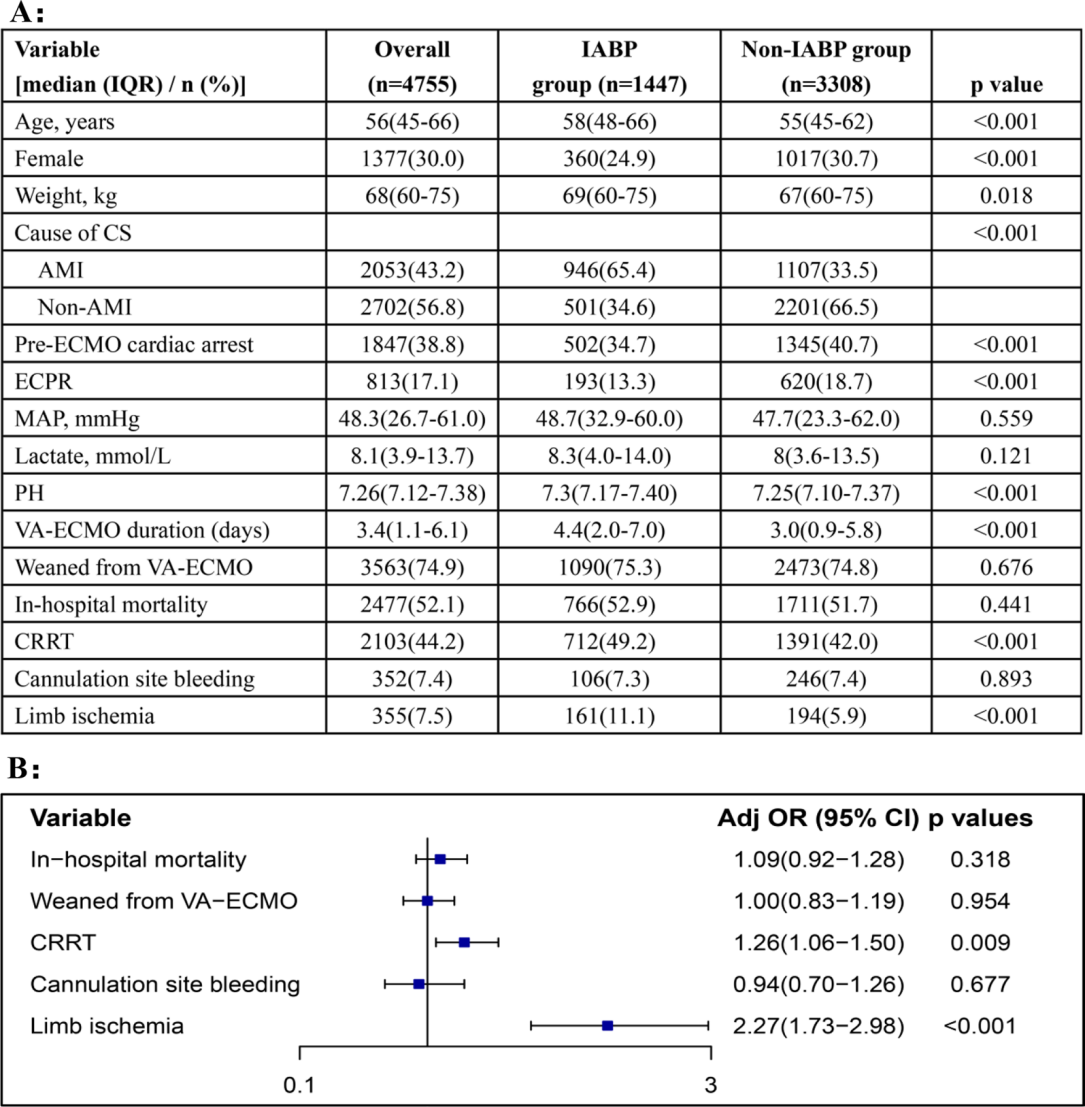
**A** Table demonstrating characteristics of patients supported with VA-ECMO stratified by IABP use. **B** Forest plot of the OR (95% CI) from multivariable logistic regression modeling examining the association of IABP and outcomes in VA-ECMO patients

The rates of in-hospital mortality (52.9% vs 51.7%, *p* = 0.441; Fig. 1B), weaning from VA-ECMO (75.3% vs 74.8%, *p* = 0.676), and cannulation site bleeding (7.3% vs 7.4%, *p* = 0.893) were similar between the IABP group and the non-IABP group. CRRT (49.2% vs 42.0%, *p* < 0.001) and limb ischemia (11.1% vs 5.9%, *p* < 0.001) were significantly more frequent in the IABP group. In multivariable logistic regression analyses, with adjustment for age, sex, weight, cause of CS, pre-ECMO CA, ECPR, mean arterial pressure (MAP), lactate, PH, and VA-ECMO duration, IABP use was associated with similar rates of in-hospital mortality (OR 1.09; 95% CI 0.92–1.28; *p* = 0.318), weaning from VA-ECMO (OR 1.00; 95% CI 0.83–1.19; *p* = 0.954), and cannulation site bleeding (OR 0.94; 95% CI 0.70–1.26; *p* = 0.677), but higher rates of CRRT(OR 1.26; 95% CI 1.06–1.50; *p* = 0.009) and limb ischemia(OR 2.27; 95% CI 1.73–2.98; *p* < 0.001).

In this large, Chinese, registry-based cohort study, IABP was not associated with lower in-hospital mortality, which was inconsistent with recent meta-analyses or observational studies [2, 3]. This association might be explained by the relatively low efficacy of IABP in LV unloading. Organ complications were main causes of hospital death in patients undergoing VA-ECMO. Previous studies have indicated that IABP significantly decreased mean cerebral blood flow during cardiac stunning [5], potentially increasing the incidence of neurologic complications. In addition, higher rates of renal failure requiring CRRT and limb ischemia were observed in the IABP group. These findings might also account for the absence of influence on in-hospital mortality in our study. Although IABP can increase coronary blood flow, the benefits of IABP were not found in AMI patients. On possible explanation was that the clinical conditions of patients in the IABP group were always more severe as compared to the non-IABP group.

The main limitation of this study is its observational design, so that even after adjusting for potential confounders, residual and unmeasured confounding cannot be ruled out. Furthermore, the majority of IABP devices were placed before VA-ECMO in our study. Thus, the majority of patients in the IABP group were escalated to VA-ECMO from IABP rather than having IABP placed at or after ECMO initiation specifically for LV unloading.

Among patients with CS treated with VA-ECMO, concomitant IABP did not reduce in-hospital mortality, but increased the incidences of CRRT and limb ischemia. Although this study does not support the use of IABP for VA-ECMO, clinicians should make decision based on the needs of patients and on their experience. Randomized clinical trials are warranted to investigate the effects of IABP use for VA-ECMO patients.

## Data Availability

The datasets used and/or analyzed during the current study are available from the corresponding author on reasonable request.

## Abbreviations

CS: Cardiogenic shock
CA: Cardiac arrest
VA-ECMO: Venoarterial extracorporeal membrane oxygenation
IABP: Intra-aortic balloon pump
LV: Left ventricular
CSECLS: Chinese Extracorporeal Life Support
ECPR: Extracorporeal cardiopulmonary resuscitation
AMI: Acute myocardial infarction
MAP: Mean arterial pressure
CRRT: Continuous renal replacement therapy
IQR: Interquartile range
